# A Case Study of Swine Wastewater Treatment via Electrochemical Oxidation by Ti_4_O_7_ Anode

**DOI:** 10.3390/ijerph192113840

**Published:** 2022-10-25

**Authors:** Hongyou Wan, Ruifeng Wang, Beibei Wang, Kehao Zhang, Huanhuan Shi, Hailong Wang

**Affiliations:** 1 School of Ecology and Environment, Zhengzhou University, Zhengzhou 450001, China; 2 Research Centre of Engineering and Technology for Synergetic Control of Environmental Pollution and Carbon Emissions of Henan Province, Zhengzhou University, Zhengzhou 450001, China; 3 College of Resources and Environmental Science, Henan Agricultural University, Zhengzhou 450002, China; 4 College of Materials Science and Engineering, Zhengzhou University, Zhengzhou 450001, China

**Keywords:** swine wastewater, electrochemical oxidation, Ti_4_O_7_, efficient removal

## Abstract

With the rapid development of breeding industry, the efficient treatment of dramatically increasing swine wastewater is gradually becoming urgent. In particular, the development of application technologies suitable for the relatively small piggeries is critical due to the time cost and space requirements of conventional biological methods. In this study, Electrochemical oxidation (EO) was selected to systematically explore the treatment performance of three different swine wastewaters by Ti_4_O_7_ anode. It was observed that the colors changed from dark brown to light yellow after 60 min treatment at 50 mA/cm^2^, and the removal rates of turbidity and suspended solids ranged from 89.36% to 93.65% and 81.31% to 92.55%, respectively. The chemical oxygen demand (COD), ammonia nitrogen (NH_3_-N) and total phosphorus (TP) of all the three swine wastewaters were simultaneously removed to a very low concentration in 120 min, especially for sample III, 61 ± 9 mg/L of COD, 6.6 ± 0.4 mg/L of NH_3_-N and 5.7 ± 1.1 mg/L of TP, which met the Discharge Standard of Pollutants for Livestock and Poultry Breeding (GB 18596-2001). Moreover, 70.93%–85.37% mineralization rates were also achieved in 120 min, confirming that EO treatment by Ti_4_O_7_ could efficiently remove the organic matters in wastewater. Excitation–emission matrix (EEM) and UV-vis spectrum characterization results further proved that aromatic compounds and macromolecules in wastewater were rapidly removed, which played important roles in the mineralization processes. The findings here provided an efficient and environment-friendly technology for swine wastewater treatment.

## 1. Introduction

In recent years, the scale of the intensive piggery industry has developed rapidly and become one of the important pillars of rural economic development [1,2]. According to the statistics released by the National Bureau of Statistics of China, more than 500 million pigs were bred annually in China [3,4], which means a dramatic increase in swine wastewater output containing abundant volumes of various contaminants, such as highly concentrated organic matter, ammonia nitrogen and phosphorus [5,6,7]. The incomplete treatment of swine wastewater by centralized wastewater treatment plants might result in adverse effects in receiving streams, which could affect the broad environmental components and pose a serious risk to the public health [8,9]. Therefore, the advanced treatment of swine wastewater is necessary to meet the limits imposed by discharge standards before being discharged to the natural environment.

Generally, biological methods can meet the requirements for effective treatment of swine wastewater with high organic loads. However, time cost and space requirements inhibit its large-scale promotion, especially for the relatively small piggeries [10]. The application of electrochemical oxidation (EO) for wastewater treatment is drawing increasing attention [11,12,13,14], and it has been regarded favorably as a next-generation water treatment technology for a few reasons. Compared with the conventional biological methods, EO can efficiently function at ambient conditions, is easy to manipulate and may be conducted automatically. In addition, EO requires no addition of chemicals, and can efficiently decompose organic pollutants by direct oxidation occurring on the anode and indirect oxidation by hydroxyl free radical, which is more powerful than the conventional advanced oxidation processes. As reported, the anode material acts an important role in the treatment performance of wastewater, including the oxidation rates and removal routes of pollutants, which is also affected by the components in wastewater [15]. Anodes including Pt [16], PbO_2_ [17] and boron-doped diamond (BDD) [16] have been investigated to treat various pollutants in water [18]. However, high cost, defective stability on performance and health risks limited its further commercial application.

Magnéli phase Ti_4_O_7_ has recently been explored as a promising anode due to its high over-potential, chemical stability and low production cost [19,20,21,22]. Previous studies found that a Ti_4_O_7_ anode can be used to treat various pollutants in wastewater including land fill leachate, phenol, dye, per- and polyfluoroalkyl substances, antibiotics, polycyclic aromatic hydrocarbons and p-nitrophenol [23,24,25,26]. However, few works on the application of Ti_4_O_7_ in the treatment of real wastewater, especially for swine wastewater, were reported, and usually, only the changes in chemical oxygen demand (COD) and total organic carbon (TOC) were quantified in EO treatment. Here, the removal of colors, turbidity, suspended solids (SS), ammonia nitrogen (NH_3_-N) and total phosphorus (TP) were also tracked, which helped to expand the application scope of EO methods. Excitation-emission matrix (EEM)and UV-vis spectrum were used to reveal the conversion of organic components during EO treatment. Furthermore, X-ray diffraction (XRD) and scanning electron microscope (SEM) characterization were also employed to analyze the stability of Ti_4_O_7_ anode in the treatment processes.

## 2. Materials and Methods

### 2.1. Collection and Analysis of Swine Wastewater

Three swine wastewater samples, including raw wastewater, biotreated effluent after anaerobic process by upflow solids reactor and effluent after secondary sedimentation tank, were named sample I, II and III, respectively, and were collected from a pig farm in Zhengzhou, Henan province, China. The analyses of colors, SS, turbidity, COD, NH_3_-N and TP were performed according to the methods set forth in the Discharge Standard of Pollutants for Livestock and Poultry Breeding (GB 18596–2001), from the Ministry of Ecology and Environment of China. TOC was determined using TOC analyzer (TOC-L CPN, CN2600, Shimadzu, Kyoto, Japan). EEM and UV scanning were conducted using a fluorescence spectrometer (F-4600, Hitachi, Tokyo, Japan) and UV-vis spectrophotometer (T6, PGENERAL, Beijing, China), respectively. The characteristics results of the wastewater used here are shown in Table 1. It was found that the major water quality indicators of the wastewater samples, such as SS and TP, greatly exceeded the discharge standard (GB 18596–2001), suggesting that the technological development of efficient treatment is needed.

### 2.2. Ti_4_O_7_ Electrode Fabrication

The Ti_4_O_7_ electrode was prepared by a spark plasma sintering (SPS) system (SPS-20T-10-IV, Shanghai Chenhua Science Technology Co., Ltd., Shanghai, China) at 1100 °C under 1 ton pressure for 20 min to sinter micron-sized Ti_4_O_7_ powder into circular sheet-like electrode (50 mm diameter, ~3.0 mm thickness). Then, the prepared electrode was polished, and activated under −2.0 V vs. SHE cathodic polarization for two hours before used.

### 2.3. Electrooxidation Experiments

The EO treatment experiments were performed in a 250 mL glass reactor in batch mode with the Ti_4_O_7_ anode (50 mm diameter) placed vertically in the middle, and two same-sized 304 stainless steel rods in parallel on both sides of the anode as cathode; the distances between anode and cathode were kept at 18 mm.

For treatment, a 100 mL wastewater sample was continuously stirred in the reactor, and a constant current density at 50 mA/cm^2^ was applied to the cell using a DC power source (DH1718E-4, Beijing Dahua Radio Instrument Company, Beijing, China). All experiments were carried out at room temperature (25 ± 1 °C). Meanwhile, the energy consumption per kg COD (*EC_COD_*, kWh/kg_COD_) was estimated by Equation (1):

(1) $$EC_{COD}=\frac{U_{cell}\times I\times t}{\left(COD_{0}-COD_{t}\right)\times V_{W}}$$

where, *U_cell_* (V) is the cell voltage, *I* (A) is the applied current, *t* (h) is the treated time, *V_W_* (L) is the volume of wastewater to be treated and *COD_0_* and *COD_t_* (in g O_2_/L) are the COD concentrations of wastewater samples at time 0 and *t*, respectively.

## 3. Results and Discussion

### 3.1. Changes in the Colors, Turbidity and SS in the EO Treatment

**Figure 1:**
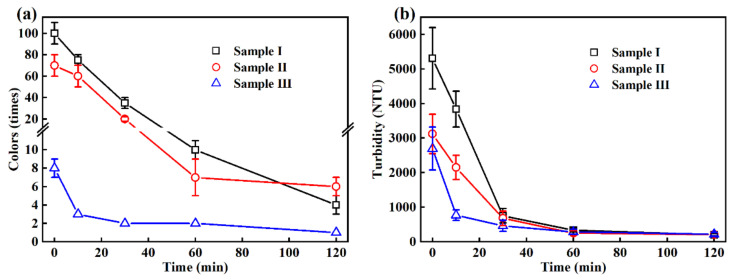
Changes in (**a**) colors and (**b**) turbidity of wastewater in EO treatment at 50 mA/cm^2^ current density.

**Figure 2:**
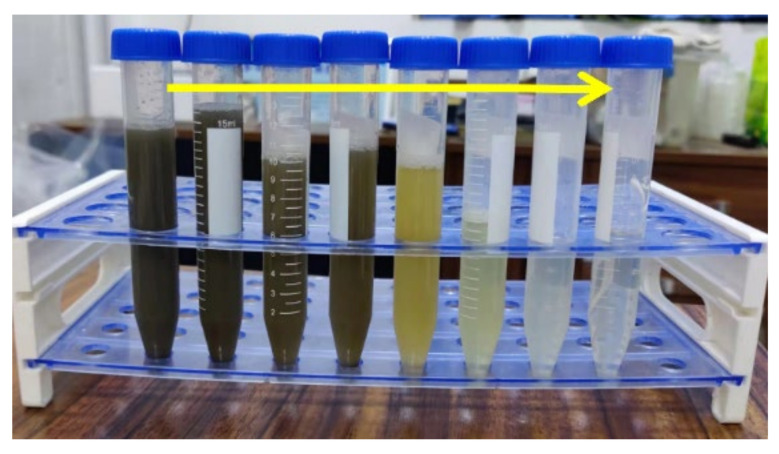
The apparent effect of EO on swine wastewater by Ti_4_O_7_ anode at 50 mA/cm^2^ current density (the solution from left to right is sample I with different treated time: 0 min, 5 min, 10 min, 15 min, 30 min, 60 min, 120 min and 180 min, respectively).

The treatment performance of wastewater was characterized using various water parameters. For the colors, it was found that the continuous removal was evident with the reaction time; for example, the colors of sample I reduced from 100 ± 10 times to about 10 ± 1 times (Figure 1a) in 60 min. As seen in Figure 2, it gradually changed from dark brown to light brown, yellow and light yellow. However, the further attenuation quickly decelerated, that is, only six times reduction occurred until 120 min. The treatment of sample II and sample III exhibited similar removal trends as shown in Figure 1a. According to the previous studies, the electrochemical hypochlorination was proposed as the main bleaching mechanism, which can make the wastewater gradually tend to be colorless and transparent by generating hypochlorous acid/hypochlorite active chlorine from chloride in wastewater (Reaction R1–R3) [27,28,29].

(R1) $$2Cl^{-}\overset{\;\;\;\;\;\;\;\;}{\to }Cl_{2}+2e^{-}$$

(R2) $$Cl_{2}+H_{2}O\overset{\;\;\;\;\;\;\;\;}{\to }HOCl+H^{+}+Cl^{-}$$

(R3) $$HOCl\overset{\;\;\;\;\;\;\;\;}{\to }ClO^{-}+H^{+}$$

The removal results of turbidity in Figure 1b showed that significant reduction was achieved in EO treatment, namely, the turbidity of sample I, sample II and sample III decreased from 5310 ± 889 NTU, 3119 ± 568 NTU and 2697 ± 622 NTU to 753 ± 211 NTU, 679 ± 208 NTU and 462 ± 163 NTU in 30 min, respectively. The reduction in light-shielding substances in wastewater benefited from the direct oxidation and indirect oxidation in EO system. It was also found that the Brownian motion of suspended particles was intensified, probably due to the thermal effect from considerable current applied in EO system, which can improve the mass transfer efficiency of pollutants between bulk solution and anode surface, accelerating the removal of components such as suspended particulates and color-causing substances which mainly contribute the turbidity in wastewater.

**Figure 3:**
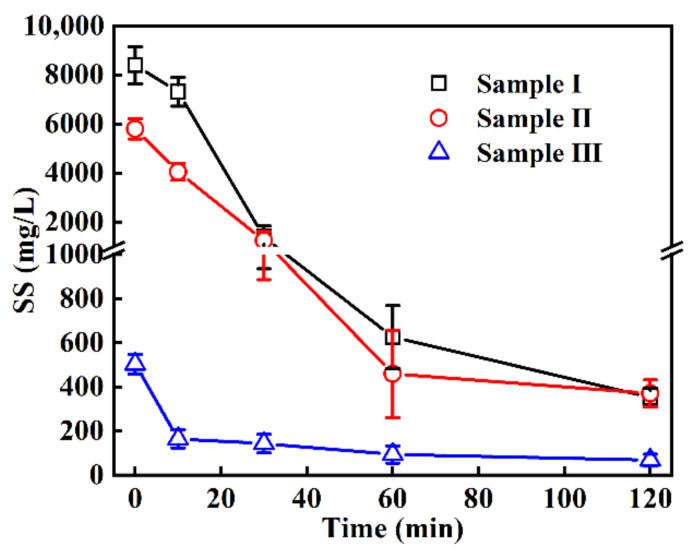
Changes in SS in wastewater in EO treatment at 50 mA/cm^2^ current density.

The SS changes were also monitored in the EO treatment process. As seen in Figure 3, the SS of sample I and sample II rapidly decreased within the initial 30 min, from 8393 ± 759 mg/L and 5794 ± 407 mg/L to 1389 ± 454 mg/L and 1246 ± 360 mg/L, respectively. Sample III with relatively lower SS also had a significant removal in EO treatment, and the treated water in 10 min quickly met the Discharge Standard (GB 18596–2001) in China (<200 mg/L). It was found that the removal curve of SS contained two distinct decreasing stages depending on the progress of the treatment; that is, a rapid decline in 0–30 min and a slow decline in 30–120 min. This is probably because the mutual agglomeration of organic substances in SS was effectively oxidized, leaving the solids containing sediment and/or some insoluble inorganic salts that were difficult to be removed. Meanwhile, it should be noted that SS could effectively weaken the convective exchange between deep end and surface water in wastewater. In particular, the consumption of dissolved oxygen during the decomposition of organic matter in SS will promote the anaerobic decomposition to deteriorate aquatic environment by the generation of hazardous gases such as hydrogen sulfide and mercaptan [30,31]. Therefore, the efficient removal of SS will help to maintain the water quality.

### 3.2. Removal of the COD and TOC in the EO Treatment

**Figure 4:**
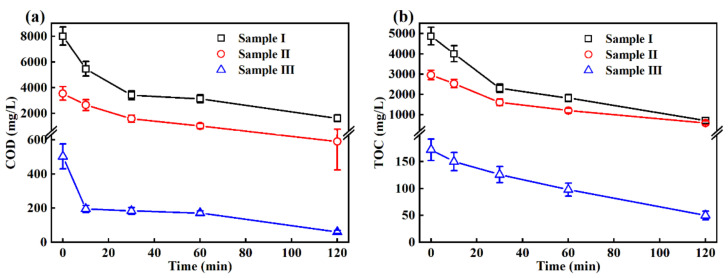
Changes in (**a**) COD and (**b**) TOC of wastewater in EO treatment at 50 mA/cm^2^ current density.

Further measurement showed that efficient removals of COD and TOC of the wastewater samples were also achieved in the EO treatment process. As shown in Figure 4a, COD of sample I decreased significantly in the initial 30 min (from 8014 ± 708 mg/L to 3415 ± 340 mg/L) during EO treatment, followed by a slower decrease to 1614 ± 265 mg/L at 120 min. For sample II and sample III, a more moderate removal trend appeared, which is probably due to easily degradable organic matters that have already been treated in the biochemical stage of wastewater treatment in the pig farm, leaving those refractory organics behind here [10].

The energy consumption (*EC_COD_*) of swine wastewater treatment was also evaluated for the two different periods (period I: 0–30 min and period II: 30–120 min), respectively, and the results are listed in Table 2. It was found that *EC_COD_* values of all the samples in period I were one order of magnitude lower than that in period II, and sample I with higher initial COD had much lower *EC_COD_* than others. Thus, EO treatment could act as an efficient method to be selected to conduct the pretreatment of high-concentration organic wastewater, to reduce the organic load of subsequent treatment units.

As shown in Figure 4b, the TOC of swine wastewater was also efficiently removed; the TOC of sample I decreased from 4875 ± 435 mg/L to 2301 ± 210 mg/L in 30 min, indicating significant mineralization occurred (52.80 ± 8.92%), while only 45.44 ± 5.44% and 26.74 ± 8.72% mineralization rates in 30 min were achieved for sample II and sample III under the same treatment conditions, respectively, which is consistent with the results of COD measurement. Greater mineralization could be achieved by prolonging EO treatment time as shown in Figure 4b.

### 3.3. Removal of the NH_3_-N and TP in the EO Treatment

NH_3_-N and TP are also two important indicators of wastewater quality. As reported, generally, high concentration of NH_3_-N and TP in wastewater could lead to eutrophication by accelerating the depletion of dissolved oxygen in receiving waterbody, making the water body become black and smelly, which posed risk to aquatic organisms and even human beings [32,33].

**Figure 5:**
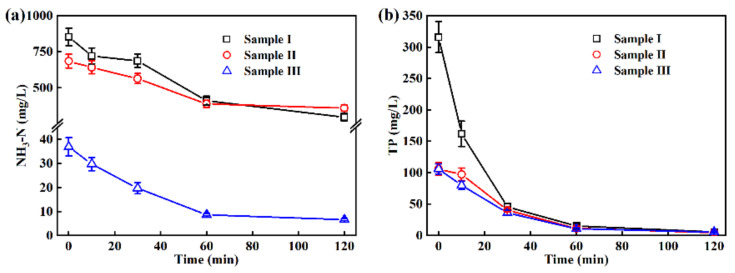
Changes in (**a**) NH_3_-N and (**b**) TP of wastewater in EO treatment at 50 mA/cm^2^ current density.

As seen in Figure 5a, the NH_3_-N concentration of sample I and sample II decreased from 853 ± 61 mg/L and 684 ± 48 mg/L to 296 ± 28 mg/L and 359 ± 22 mg/L in 120 min of EO treatment, respectively. The same conclusion should be drawn with COD and TOC results, that is, EO treatment by Ti_4_O_7_ anode is more suitable for the pretreatment of wastewater containing high-concentration NH_3_-N. Previous studies have found that the indirect oxidation by HClO generated in EO system and deprotonation by hydroxyl ions on anode contributed to the removal of ammonia [34,35,36,37,38].

The main sources of TP in water are phosphorus-containing detergents, chemical fertilizers and organophosphorus pesticides, which have a more stringent related discharge standard for the wastewater treatment process (<8 mg/L). In this study, the TP of samples I, II and III were efficiently removed (Figure 5b), and the concentration in treated samples at 120 min (5.5 ± 0.7 mg/L, 4.8 ± 0.4 mg/L, and 5.7 ± 1.1 mg/L, respectively) met the related discharge standard. Based on the literature, the removal mechanism of TP in wastewater might be attributed to the transformation of organic phosphorus into a more easily removable form of inorganic phosphorus in EO treatment, which further reacted with Ca^2+^ and/or Mg^2+^ to form insoluble precipitates [39,40,41,42]. In addition, the removal might also be caused by that Fe^2+^ in wastewater was oxidized to Fe^3+^ which reacted with phosphate radicals to form FePO_4_ [43,44,45].

### 3.4. Changes in Excitation-Emission Matrix and UV-Vis Absorbance in EO Treatment

**Figure 6:**
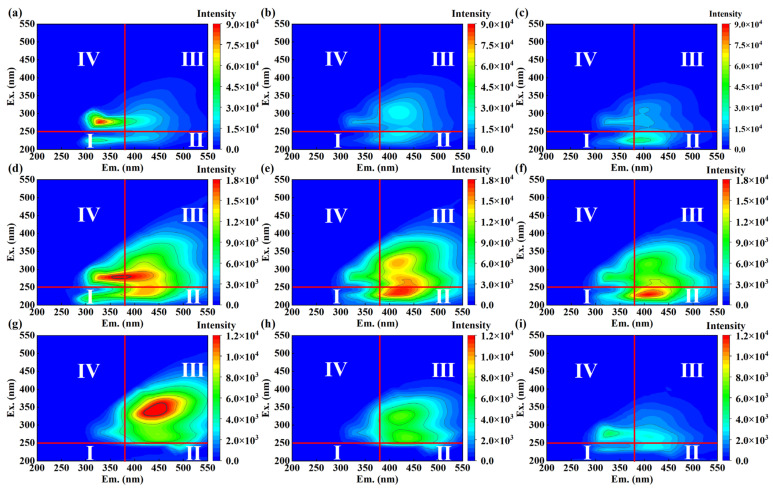
EEM of swine wastewater treated by Ti_4_O_7_ anode at 50 mA/cm^2^ current density: (**a**–**c**) are pristine sample I, 30 min treated sample I and 120 min treated sample I, respectively. (**d**–**f**) are pristine sample II, 30 min treated pristine sample II and 120 min treated sample II, respectively. (**g**–**i**) are pristine sample III, 30 min treated sample III and 120 min treated sample III, respectively.

EEM has been widely used in organic wastewater characterization [46,47]. The fluorescence spectra of swine wastewater samples before and after EO treatment were analyzed to investigate the removal mechanism of organic matter. As reported, the excitation (Ex.) and emission (Em.) wavelength of EEM were divided into four different regions: aromatic proteins region (I) at Ex./Em. of <250 nm/<380 nm, fulvic acid-like region (II) Ex./Em. of <250 nm/>380 nm, soluble microbial byproduct-like region (III) Ex./Em. of >250 nm/<380 nm and humic acid-like region (IV) Ex./Em. of >250 nm/>380 nm [48]. As shown in Figure 6a, a high intensity fluorescence peak of untreated sample I was located at Ex./Em. = 280 nm/330 nm, which was reported as the region of humic acid-like moieties. The main fluorescence peaks of sample II and sample III were identified as Ex./Em. = 280 nm/370 nm and Ex./Em. = 340 nm/445 nm, respectively. It was found that the significant weakening of fluorescence intensities for the three samples was observed with treatment time, indicating that the organic matter of samples was partially degraded by EO treatment. Additionally, fulvic acid-like compounds might be generated from the oxidation of SMPs by comparison of results in Figure 6e,f.

The UV-vis scanning was also adopted to evaluate the changes in swine wastewater samples in treatment process, and the results including UV_254_, UV_410_ and UV_250/365_ are listed in Table 3. We found that the UV_254_ and UV_410_ values of wastewater samples greatly decreased after EO treatment, indicating that the aromatic compounds and macromolecules were effectively decomposed in EO treatment process. Moreover, the increase in UV_250/365_ ratio suggested that humic acid-like substances have been well removed [49].

### 3.5. Stability Characterization of Anode

**Figure 7:**
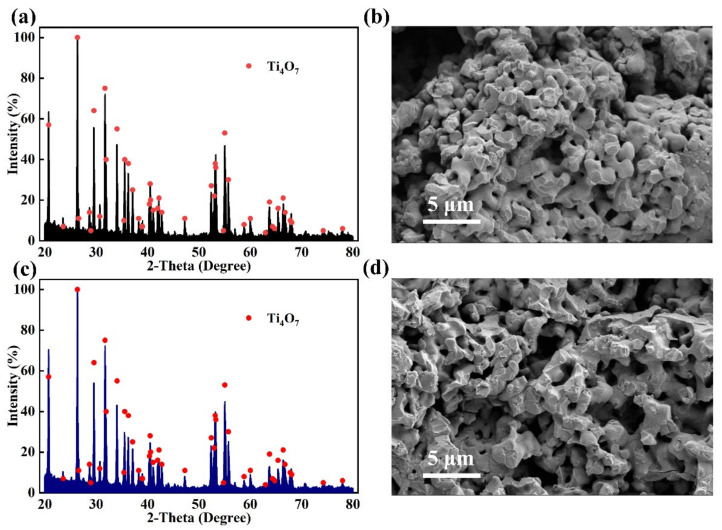
The (**a**) XRD and (**b**) SEM of Ti_4_O_7_ anode without treatment, (**c**) XRD and (**d**) SEM of Ti_4_O_7_ anode after several cyclic treatment.

The XRD spectrum of the anode in Figure 7a confirms that Ti_4_O_7_ electrode was successfully prepared. As seen in the SEM image of anode (Figure 7b), the Ti_4_O_7_ electrode has extensive interconnecting porous structures that helps the efficient treatment of pollutants in the wastewater [50]. The XRD and SEM analyses of the anode after several cyclic treatment were also performed (Figure 7c,d). It was found that the phase composition and surface morphology of Ti_4_O_7_ anode did not change significantly. In addition, the triplicate experiments for each treatment always exhibited satisfactory treatment effect in swine wastewater treatment, confirming the durability of Ti_4_O_7_ electrode prepared in this study. The stability of Ti_4_O_7_ in different reaction conditions has also been proved in the previous studies [51,52,53]. Therefore, it is feasible in swine wastewater treatment to use EO methods by Ti_4_O_7_ anode.

## 4. Conclusions

In this study, the electrochemical oxidation behaviors of swine wastewater with a large amount of organic matter by Ti_4_O_7_ anode were performed and characterized by multiple indicators. Our results demonstrate that colors in sample I changed from dark brown to light yellow in 60 min, and turbidity and SS were quickly removed in EO treatment. The chemical indicators of COD, NH_3_-N and TP in all three wastewater samples were efficiently removed. Meanwhile, 70.93–85.37% mineralization rates were achieved for the three swine wastewater samples in 120 min, which was further demonstrated according to the EEM and UV-vis spectra results that macromolecules, including humic acid-like substances, and SMPs were removed in EO treatment. The results here proved that EO treatment method by Ti_4_O_7_ anode is a reliable and promising alternative for the treatment of high concentration polluting wastewaters, such as swine wastewater.

## Figures and Tables

**Table 1:** Characterization of swine wastewater samples.

Water Parameters	Sample I	Sample II	Sample III	Discharge Standard (GB 18596–2001)
Colors (times)	100 ± 10	70 ± 10	8 ± 1	-
Turbidity (NTU)	5310 ± 889	3119 ± 568	2697 ± 622	-
SS (mg/L)	8393 ± 759	5794 ± 407	503 ± 45	200
COD (mg/L)	8014 ± 708	3550 ± 532	503 ± 73	400
TOC (mg/L)	4875 ± 435	2958 ± 235	172 ± 20	-
NH_3_-N (in N, mg/L)	853 ± 61	684 ± 48	37 ± 3.8	80
TP (in P, mg/L)	316 ± 25	106 ± 10	106 ± 8.2	8

**Table 2:** Energy consumption of swine wastewater via EO treatment by Ti_4_O_7_ anode at 50 mA/cm^2^ current density.

*EC_COD_* (kWh/kg_COD_)	Sample I	Sample II	Sample III
period I	8.15	20.42	125.39
period II	70.79	130.37	1024.39

**Table 3:** UV-vis indicators of swine wastewater by Ti_4_O_7_ anode at 50 mA/cm^2^ current density.

Wastewater		UV_254_	UV_410_	UV_250/365_
Sample I	Pristine	0.627	0.165	2.947
	After 30 min treatment	0.409	0.082	3.523
	After 120 min treatment	0.147	0.013	7.533
Sample II	Pristine	0.547	0.148	2.824
	After 30 min treatment	0.268	0.047	3.827
	After 120 min treatment	0.145	0.011	7.800
Sample III	Pristine	0.778	0.184	2.979
	After 30 min treatment	0.466	0.067	4.772
	After 120 min treatment	0.290	0.052	4.451

## Data Availability

Not applicable.
